# Tattoo toxicology, an upcoming complex scientific issue

**DOI:** 10.1007/s00204-020-02822-2

**Published:** 2020-06-30

**Authors:** Hermann M. Bolt, Jan G. Hengstler

**Affiliations:** Leibniz Research Centre for Working Environment and Human Factors at TU Dortmund (IfADo), Adeystr. 67, 44139 Dortmund, Germany

A Guest Editorial in Archives of Toxicology by Schreiver and Luch (2016) has pointed to the new field of tattoo toxicology and to existing data gaps. A global trend is now noted towards larger and more colourful tattoos (Bagot 2020). Yet, tattoo legislation in Europe is still based on an exposure scenario of placing the product on top of the skin, rather than right into the middle of living tissue beneath the epidermal skin barrier (Schreiver and Luch 2016).

## Toxic effects of tattooing

From a consumer’s aspect, there is a notable increase in the incidence of “itchy tattoos” (Kluger 2019a). From a clinical point of view, hypersensitivity reactions to tattoo inks, mostly elicited by specific colorants, are the most common tattoo complications, and these are unpredictable (Kluger 2019b). Hypersensitivity may appear after a latency of several months or even years; the state is mostly chronic, and it is resistant against treatment with corticoids. Moreover, epicutaneous patch testing with specific tattoo inks is usually negative, because the reaction is due to hapten formation within the inner skin, by combination with a protein and/or by the involvement of degradation products (Kluger 2019b; Bagot 2020).

Considerable progress has been made in tattoo pigment analysis. For identification of 36 specific organic pigments in tattoo inks, Schreiver et al. (2016) described an analytical method in this journal, which is based on pyrolysis–gas chromatography/mass spectrometry (py-GC/MS). On this basis, there is a perspective to differentiate between more or less harmful pigments.

A pivotal point of the toxicological discussion is the potential carcinogenicity of tattoo inks. At present, skin cancers on tattoos are considered so far as coincidental, except for keratoacanthomas on red tattoos (Swigost et al. 2019; Kluger 2019b). But irrespective of the lack of epidemiological data, genotoxic and carcinogenic primary aromatic amines or polycyclic aromatic hydrocarbons have been detected as impurities of tattoo inks (Schreiver and Luch 2016).

Another aspect, commented by Schreiver and Luch (2020) in the February issue of this journal, is the introduction of metal debris from tattoo needles containing high amounts of chromium and nickel into the skin. Especially the presence of nickel as a powerful contact allergen is a matter of toxicological concern.

## Toxicity of tattoo removal

Laser irradiation is the most common way to remove unwanted tattoos. Nowadays, primarily organic pigments are used for brilliant colour shades, but such laser irradiation of organic pigments has been shown to produce carcinogenic compounds, e.g. 3,3′-dichlorobenzidine (Hering et al. 2018). Hence, a new avenue of research is the generation of toxic fragments from tattoo inks upon tattoo removal procedures.

This issue of Archives of Toxicology presents two contributions to this topic.

(i) Bauer et al. (2020) report on the products of laser treatment of a specific green tattoo ink. The ink characterisation was carried out by IR, UV–Vis, EDX spectroscopies and SEM imaging. This revealed the presence of pigment PG7, rather than PG36 as reported on the bottle label, along with non-fully halogenated analogues. The ink morphology was an extended sheath with embedded grains. Laser treatments performed on both dried and extracted inks. The products were analysed by gas chromatography–mass spectrometry, UV–Vis spectroscopy, SEM imaging and dynamic light scattering. The outcome was a complex fragmentation pattern that depended both on the solvent and on the initial aggregation state. The fragment compounds were toxic at various degrees, according to current classification labelling and packaging (CLP) regulations.
(ii) Hering et al. (2020) establish a reconstructed full-thickness skin model with tattoo pigments, *TatS*. This new and promising model emulates healed tattooed human skin. It underlines the advantages of 3D over traditional 2D cell culture systems. The methodological approach might be important for further research on the toxicology of tattooing, including used pigments and their destruction for tattoo removal.

## Regulatory issues

In the February issue of this journal Giulbudagian et al. (2020) reviewed current regulatory considerations in the European Union. An exemplary initiative for improving the safety of tattooing is warranted. On the one hand, the compilation of market surveillance data provides knowledge on hazardous substances present in tattoo inks. On the other hand, clinical data from patients now enable the correlation of adverse reactions with certain defined substances.

Nevertheless, the assessment of risks remains a challenge, owing to knowledge gaps on biokinetics of highly complex inks and their degradation products. Giulbudagian et al. (2020) point to strategies for regulating substances in tattoo inks in the light of potential future restrictions in the frame of the European REACH regulation.
